# Attenuation of Radiation-Induced Lung Injury by Hyaluronic Acid Nanoparticles

**DOI:** 10.3389/fphar.2020.01199

**Published:** 2020-08-12

**Authors:** Anna Lierova, Jitka Kasparova, Jaroslav Pejchal, Klara Kubelkova, Marcela Jelicova, Jiri Palarcik, Lucie Korecka, Zuzana Bilkova, Zuzana Sinkorova

**Affiliations:** ^1^Department of Radiobiology, Faculty of Military Health Sciences, University of Defence, Hradec Kralove, Czechia; ^2^Department of Biological and Biochemical Sciences, Faculty of Chemical Technologies, University of Pardubice, Pardubice, Czechia; ^3^Department of Toxicology and Military Pharmacy, Faculty of Military Health Sciences, University of Defence, Hradec Kralove, Czechia; ^4^Department of Molecular Pathology and Biology, Faculty of Military Health Sciences, University of Defence, Hradec Kralove, Czechia; ^5^Institute of Environmental and Chemical Engineering, Faculty of Chemical Technology, University of Pardubice, Pardubice, Czechia

**Keywords:** radiation, lung, radiation fibrosis, hyaluronic acid, nanoparticles

## Abstract

**Purpose:**

Therapeutic thorax irradiation as an intervention in lung cancer has its limitations due to toxic effects leading to pneumonitis and/or pulmonary fibrosis. It has already been confirmed that hyaluronic acid (HA), an extracellular matrix glycosaminoglycan, is involved in inflammation disorders and wound healing in lung tissue. We examined the effects after gamma irradiation of hyaluronic acid nanoparticles (HANPs) applied into lung prior to that irradiation in a dose causing radiation-induced pulmonary injuries (RIPI).

**Materials and Methods:**

Biocompatible HANPs were first used for viability assay conducted on the J774.2 cell line. For *in vivo* experiments, HANPs were administered intratracheally to C57Bl/6 mice 30 min before thoracic irradiation by 17 Gy. Molecular, cellular, and histopathological parameters were measured in lung and peripheral blood at days 113, 155, and 190, corresponding to periods of significant morphological and/or biochemical alterations of RIPI.

**Results:**

Modification of linear hyaluronic acid molecule into nanoparticles structure significantly affected the physiological properties and caused long-term stability against ionizing radiation. The HANPs treatments had significant effects on the expression of the cytokines and particularly on the pro-fibrotic signaling pathway in the lung tissue. The radiation fibrosis phase was altered significantly in comparison with a solely irradiated group.

**Conclusions:**

The present study provides evidence that application of HANPs caused significant changes in molecular and cellular patterns associated with RIPI. These findings suggest that HANPs could diminish detrimental radiation-induced processes in lung tissue, thereby potentially decreasing the extracellular matrix degradation leading to lung fibrosis.

## Introduction

Despite the many great advances in the field of radiotherapy, radiation toxicity remains a serious complication in patients receiving such therapy. The lung, in particular, is one of the organs most critically limited by the development of radiation-induced pulmonary injuries (RIPI). The proportion of patients susceptible to RIPI after radiotherapy is as high as 20% (Hanania et al., 2019; Giuranno et al., 2019). The currently accepted model of RIPI has been described as a continuous and multicellular process beginning immediately after insult. Three phases can be recognized within RIPI and also distinguished temporally, these being the latent period, pneumonic phase, and late-fibrotic phase. After irradiation, initial damage induces expression of multiple cytokines and growth factors in specific cell types within lung tissue. This is followed by activation of various signaling pathways and results in the development of subsequent pathophysiological processes. The first manifestation of RIPI is radiation pneumonitis (RP), an acute inflammatory process. RP is characterized by the recruitment of diverse immune cells into the tissue; expression of diverse cytokines, chemokines, and adhesion molecules; and, as a result, edema of the interstitium and alveolar spaces (Schaue et al., 2012; Lierova et al., 2018). The terminating phase, radiation fibrosis (RF), is driven primarily by accumulation of fibroblasts and myofibroblasts, which extensively produce collagen and extracellular matrix (ECM) proteins causing tissue and impaired organ function. Except fibroblast, myofibroblasts may also derive from circulating bone marrow-derived progenitor cells known as fibrocytes or from trans-differentiated epithelial cells (alveolar pneumocytes type I and type II) *via* epithelial–mesenchymal transition (Jang et al., 2013; Lierova et al., 2018; Jin et al., 2020). Recently, the endothelial-to-mesenchymal transition of vascular endothelial cell has been identified also as contributing factor to development of RF (Choi et al., 2015). From another point of view, RF may be perceived as insufficient wound healing characterized by extensive deposition of connective tissue that is followed by destruction of the lung parenchyma to form fibrotic lesions (Straub et al., 2015). Both concepts emphasize the role of transforming growth factor β (TGF-β) and its signaling, but accumulating evidence points to the role of ECM components in the fibrotic process (Wegrowski et al., 1992; Bensadoun et al., 1996; Albeiroti et al., 2015; Collum et al., 2019).

One of the main ubiquitously and naturally occurring moieties in ECM is hyaluronic acid (HA). This linear and negatively charged polysaccharide is widely distributed in many organs and tissues. In lung, HA is present from the time of fetal organ development in the alveolar region, in the basement of bronchiolar and bronchial epithelium, and after insult (bleomycin or hyperoxia) also in perivascular vessels, an area around larger pulmonary arteries. HA plays several important roles in the organization and modification of ECM by binding with cells and other components through specific and nonspecific interaction. It is also regarded as an important signaling molecule and a regulator of inflammatory responses and tissues healing after insult (Singleton and Lennon, 2011; Lauer et al., 2015). HA is unique among other active molecules in that its biological effects depend upon HA fragment size (Cyphert et al., 2015). Under physiological conditions, ECM consists of high-molecular-weight (HMW-HA) HA with an average size range of 1–5 × 10^6^ Da. In alveoli, HA is synthesized on cellular surface by hyaluronan synthase 2 (HAS2), and particularly by type II pneumocytes, and it is required for optimal cell survival, maintenance of homeostasis, and self-renewal in healthy lung tissue. The molecule interacts through CD44 receptor on alveolar macrophages (Johnson P. et al., 2018).

HA plays several important roles in the organization and modification of ECM by binding with cells and other components through specific and nonspecific interaction. It is also considered to be an important signaling molecule and regulator of inflammatory response and tissues healing after insult (Dicker et al., 2014). Under certain pathological conditions, including ionizing radiation (IR) and presence of radical oxygen species, HA is a doubled-edge sword in lung tissue. HA is degraded into low-molecular-weight (LMW-HA) bioactive fragments, ranging in size from 2 × 10^4^ to 2 × 10^6^ Da, with strong pro-inflammatory properties. These particles even may serve as danger-associated molecular patterns and endogenous danger molecules, and they may initiate and perpetuate a noninfectious inflammatory response (Fallacara et al., 2018). Animal models as well as human studies have shown that HA levels rapidly increase in the lung during inflammation and peak with maximum leukocyte infiltration. The tissue homeostasis is restored when HA concentrations decline and its levels return to baseline (Nilsson et al., 1990; Bray et al., 1991; Dentener et al., 2005; Liang et al., 2011). Nevertheless, LMW-HA fragments may strongly augment the inflammatory response. Although LMW-HA interacts *via* CD44, its signaling supports inflammatory cell recruitment, including of T cells, to the injury site (Avenoso et al., 2020). Data from CD44^-/-^ deficient mice have shown increased accumulation of LMW-HA, enhanced pro-inflammatory gene expression, and unremitting inflammation in the lung tissue after intratracheal bleomycin (Teder et al., 2002) or lipopolysaccharide (Liang et al., 2007) treatment, thereby suggesting that CD44 is crucial for successful renewal of homeostasis.

On the other hand, exogenous administration of HA has shown great therapeutic potential. HMW-HA exerts competitive negative feedback on LMW-HA. The molecular weight of HA also affects its affinity for CD44 receptor, and the ratio of LMW-HA/HMW-HA may be important for maintenance of ECM integrity and the final extent of lung injury. This effect has been demonstrated by multiple HMW-HA applications for diverse lung injury modalities, including elastase-induced emphysema, allergic airway inflammatory lung injury, and bleomycin-induced lung damage in mice (Jiang et al., 2005; Cantor, 2007; Johnson C. G. et al., 2018), as well as lipopolysaccharide-induced sepsis and acute lung injury induced by inhalation of fine particulate matter in rats (Liu et al., 2008; Xu et al., 2018). In humans, inhaled HMW-HA improves homeostasis in cystic fibrosis and protects against disease exacerbations in patients with chronic bronchitis (Venge et al., 1996; Lamas et al., 2016). In recent years, HA has attracted attention as a polymer suitable for drug carrier systems because it can assemble into nanoparticles (NPs) (Hussain et al., 2016; Jeannot et al., 2018).

In the present study, we focus on HA assembled into NPs (HANPs) by intramolecular cross-linking. Two different diameters (123.6 and 86.58 nm) were prepared. The properties of the prepared HANPs were first evaluated *in vitro*. Subsequently, HANPs were administered before irradiation and their effects on the acute and chronic phases of RIPI were analyzed.

## Material and Methods

### *In Vitro* Experiments: Nanoparticles Preparation, Characterization, and Irradiation

#### Simple Cross-Linking Procedure

Hyaluronic acid sodium salt from Streptococcus equi (mol wt 1.5–1.8 MDa, ≤1% protein), adipic acid dihydrazide (AAD), 1-ethyl-3-(3′-dimethylaminopropyl)carbodiimide (EDC), and cellulose dialysis tubing (12,400 Da) were purchased from Sigma-Aldrich (St. Louis, MO, United States). All other chemicals were supplied by Lach-Ner (Neratovice, Czechia) at reagent grade. Hyaluronic acid sodium salt was dissolved in deionized water (prepared using TKA Smart2Pure, Thermo Fisher Scientific, Waltham, MA, United States) to create a 2.5 mg/ml solution. To prepare 5 mg of HANPs, 2 ml of HA solution was used and afterwards 3.4 ml of acetone was added to the solution (15 min incubation, gentle stirring). After the addition of EDC [2.5 mg/75 μl ultrapure water (prepared by TKA Smart2Pure)] and AAD (2.5 mg/75 μl), acetone was added in a stepwise manner (2.03 ml per step) in three repetitions. The total acetone/water ratio was set to 279/80 (w/w) and surplus acetone was then removed by evaporation at 60°C with following dialysis against 0.9% NaCl solution.

#### Double Cross-Linking Procedure

The cross-linking procedure was repeated using the nanoparticles prepared as described above. After the evaporation of surplus acetone, the addition of EDC (2.5 mg/75 μl ultrapure water) proceeded immediately. Following the same procedure as described above, acetone was again added in a stepwise manner. Finally, the procedure was completed by evaporation of surplus acetone and dialysis against 0.9% NaCl solution. Parameters of the prepared nanoparticles (0.25 mg of HANPs in 1.5 ml 0.9% NaCl) were monitored using dynamic light scattering (DLS; NanoZS, Malvern Instrument, United Kingdom) at 25°C in 0.9% NaCl.

#### Irradiation

The stability of both types of prepared nanoparticles was investigated. HANPs in concentration 1 mg/ml in 0.9% saline solution were irradiated with a 17 Gy dose. The same dose was later used for thorax irradiation during the *in vivo* experiments. HANPs’ properties were analyzed by DLS spectrophotometry prior to irradiation and then 1 day, 1 week, as well as 1 and 2 months after irradiation to assess their stability. The irradiation procedures used a Chisotron ^60^Co source (Chirana, Prague, Czechia) at a dose rate of 0.32 Gy/min with a target distance of 1 m.

### Viability Assay

J774.2 adherent murine macrophages (ATCC^®^, Manassas, VA, United States) were seeded (7,500 cells/well) on 96-well plates and cultured in 90 μl of Dulbecco’s Modified Eagle’s Medium with high glucose concentration (DMEM/GlutaMAX™, Gibco, Grand Island, NY, United States) supplemented with 10% fetal bovine serum and 1% antibiotics (150 UI/ml penicillin, and 50 mg/ml streptomycin; all from Sigma-Aldrich) for 24 h at 37°C and 5% CO_2_. After incubation, 10 μl of HANPs were dissolved in DMEM without supplements and added to the final concentration ranging from 1 μg/ml to 2 mg/ml. Five replicates were measured per each concentration, and DMEM without supplements was used as negative control group. After 24 h of incubation, cell viability was determined using 4-[3-(4-iodophenyl)-2-(4-nitro-phenyl)-2H-5-tetrazolio]-1,3-benzene sulfonate dye (WST-1, Roche Diagnostics GmbH, Mannheim, Germany). For that determination, 50 μl of WST-1/phosphate-buffered saline (1:4) to final concentration of 0.5 mg/ml was added to each well and plates were incubated for 3 h (37°C, 5% CO_2_). Plates were gently mixed and then measured using a Spectronic Helios Gamma microplate reader (Thermo Fisher Scientific) at wavelength 450 nm. WST viability test was performed three times to ensure reproducibility.

In the subsequent experiment, the J774.2 cell line was pre-incubated with HANPs of the same sizes and concentration range for 2 h or 30 min. After incubation, the cells were exposed to a single dose of 4 Gy. Viability was measured 24 h after irradiation using the WST-1 assay, as described above.

### *In Vivo* Experiments: Animals and Irradiation

All experimental animal protocols proceeded under supervision of the Ethics Committee (Faculty of Military Health Sciences, Hradec Kralove, Czechia). Female C57Bl/6 mice aged 12–14 weeks (Velaz, Unetice, Czechia) were kept in an air-conditioned room (22 ± 2°C and 50 ± 10% relative humidity, 12 h light/dark cycle) and allowed access to standard food and tap water *ad libitum*.

In conducting an experiment, mice were randomly divided into four groups. Prior to irradiation, all animals were anesthetized with a combination of Narketan (0.5 ml; Vetoquinol, Prague, Czechia), Rometar (0.16 ml; Bioveta, Ivanovice na Hane, Czechia), and physiological solution (2 ml; B. Braun Melsungen AG, Melsungen, Germany) by intramuscular injection. The first group (control, n = 18) was sham-treated and nonirradiated. Mice from the second group (n = 32) were only irradiated but without application of HANPs. The third and fourth groups (both n = 32) received HANPs of sizes 86.58 nm and 123.6 nm, respectively, by intratracheal instillation. The volume of HANPs was 50 µl in final concentration of 0.5 mg/ml and was instilled 30 min before irradiation. The anesthetized animals were kept in a Plexiglas box (VLA JEP, Hradec Kralove, Czechia) and received a single dose of irradiation (17 Gy, 0.30 Gy/min, 1 m) to the thoracic region. A local thoracic irradiation was performed in a jig. Head and abdomen were shielded with lead bricks 10 cm thick layer to reduce the dose to surrounding organs.

### Tissue Collection and Sample Preparation

Tissue samples were collected on days 113, 155, and 190 post-irradiation, which time points correspond with the periods of significant morphological and/or biochemical alterations of RIPI (Jackson et al., 2010). Six mice per group were euthanized by narcotic gas overdosing at each time point. Directly after euthanasia, venous peripheral blood was drawn from the heart into heparinized syringes (BD Bioscience, Franklin Lakes, NJ, United States) for determination of absolute cell counts and flow cytometry analysis. Additionally, the left lung was perfused with cold phosphate-buffered saline and divided into two parts for cytokine profiling and for flow cytometry. The right lung was dissected and fixed in 10% neutral buffered formalin (Bamed s.r.o., Ceske Budejovice, Czechia) for histological analysis.

### Absolute Blood Count and Flow Cytometry Analysis of Peripheral Blood

The heparinized blood from each animal was divided into two parts. A volume of 150 µl was designated for blood count and measured using an ABX Pentra 60 C+ hemoanalyzer (Horiba, Kyoto, Japan). The rest of the blood (200–300 µl) was lysed using EasyLyse™ (Dako, Glostrup, Denmark). The amount of cells in suspension was as determined by Turk’s solution (2% acetic acid; Sigma-Aldrich) using a hemocytometer chamber. Cells (5 × 10^5^/100 µl) were marked with two panels of monoclonal antibodies. The first panel was delineated to detect T, B, and NK lymphocytes (CD3ϵ, CD4, CD8, CD19, and NK1.1). The second panel was designed for determination of monocytes and neutrophils (CD11b, F4/80, Ly6C, and Ly6G). The following monoclonal antibodies with fluorochromes for flow cytometry analysis were purchased from BioLegend (San Diego, CA, United States): anti-mouse CD3ϵ–FITC, CD4–BV421, CD19–PE, CD11c–BV421, CD45–APC/Fire™750, F4/80–PE, and from BD Bioscience: CD8–PECy7, CD11b–BV510, Ly6C–FITC, Ly6G–PECy7, and NK1.1–APC.

### BCA Protein Assay and Enzyme-Linked Immunosorbent Assay (ELISA) of Cytokines

A sample of the lung determined for protein and cytokine profiling was immediately placed into tubes on ice, weighed, then frozen at −80°C until further analysis. Prior to analysis, the frozen lung tissue was thawed on ice, minced, then lysed with lysis buffer (10 mM TRIS-HCl/pH 8, 150 mM NaCl, 1% Triton X-100, 10% glycerol, 5 mM EDTA, and 1 mM Na_3_VO_4_) and protease inhibitor cocktail (Roche Diagnostics GmbH). Volume of lysis buffer varied depending on the weight of lung tissue. Each sample was then homogenized using an Ultra Turrax T8 tissue homogenizer (IKA, Werke, Germany) and incubated for 1 h at 4°C while rotating. The lysates were sonicated at 4°C and centrifuged. Supernatants were collected and aliquoted for determination of total protein and cytokine concentrations.

Protein concentration was measured using the Pierce™ BCA Protein Assay Kit (Sigma-Aldrich). Protein level of each sample was measured in triplicate according to the manufacturer’s instructions. Briefly, 10 µl samples were added to 190 µl of working reagent and incubated for 30 min at 37°C. Absorbance was then measured at 562 nm using the Spectronic Helios Gamma microplate reader. The actual protein concentrations were calculated from the standard curves generated by calibrator diluents.

Aliquots of supernatants from each animal in the group were pooled together and cytokine profiles were determined from the group sample. Commercially available ELISA kits were used to quantitate mouse cytokines. Cytokine kits for interleukin-1 β (IL-1β), IL-6, CCL2 (MCP-1), CCL4 (MIP-1), CXCL1 (KC), matrix-metalloproteinases-2 (MMP-2), and proMMP-9 (all from LifeSpan BioSciences, Seattle, WA), and TGF-β1 (RayBiotech, Norcross, GA) were performed according to the manufacturers’ protocols in triplicates and quantified by comparison with a standard curve using the Spectronic Helios Gamma microplate reader.

### Flow Cytometry Analysis of Lung Tissue

The part of the lung tissue intended for flow cytometry analysis was placed into a Petri dish with Iscove’s Modified Dulbecco’s Medium (IMDM, Gibco) without fetal bovine serum, weighed, minced into small chunks, and enzymatically digested in digestion medium (2 ml IMDM, 2 mg/ml collagenase type IV, and 50 U/ml deoxyribonuclease type IV; all obtained from Sigma-Aldrich) at 37°C for 45 min. After digestion, cells were filtered through a strainer (100 μm; Roche Diagnostics GmbH) to remove tissue debris. The single cell suspensions thus obtained were subsequently centrifuged, counted in a hemocytometer chamber after dilution with Turk’s solution (2% acetic acid), and resolved in phosphate-buffered saline for the next analysis. Cell suspension was first processed using Fcγ receptor blocking solution with purified CD16/32 (eBioscience, San Diego, CA). Subsequently, cell suspension (5 × 10^5^ cells/100 µl) was marked with the same two panels of monoclonal antibodies stated above. For better analysis of leukocytes, a common leukocytes marker, CD45, was added to both panels and CD11c was added to the second panel for the detection of alveolar macrophages.

### Histology

Formalin-fixed lungs were embedded into paraffin (Paramix, Holice, Czechia) and tissue sections 5 μm thick were cut using a Model SM2000 R microtome from Leica (Heidelberg, Germany). Staining with hematoxylin and eosin (H&E; Merck, Kenilworth, NJ, United States) was performed to determine the air/tissue ratio. To detected collagen fibers in lung parenchyma the histological slides were stained by picrofuchsin using Van Gieson method (DiaPath, Martinengo, Italy). Analysis of stained tissues section was done on a BX-51 microscope equipped with a DP-72 camera (both from Olympus, Tokyo, Japan) and the Image-Pro 5.1 computer image analysis system (Media Cybernetics Inc., Bethesda, MD, United States) at 200× magnification.

### Data Analysis

All data are expressed as mean ± 2× standard error of the mean (SEM), except that the results of cytokine concentration are expressed as mean ± 2× standard deviation (SD). Statistical analysis was performed by one-way ANOVA with *post hoc* Student’s *t*-test, Tukey’s and Fisher’s least significant difference (LSD) tests (WST-1 test and cytokines) or Kruskal–Wallis test with *post hoc* Mann–Whitney *U* test (total protein, flow cytometry, and histology) using STATISTICA 12 software (StataCorp, College Station, TX, United States). Differences were considered significant at *p* ≤ 0.05.

## Results

### Hyaluronic Acid Nanoparticles Stability After Irradiation and Effect on Cell Viability

**Figure 1:**
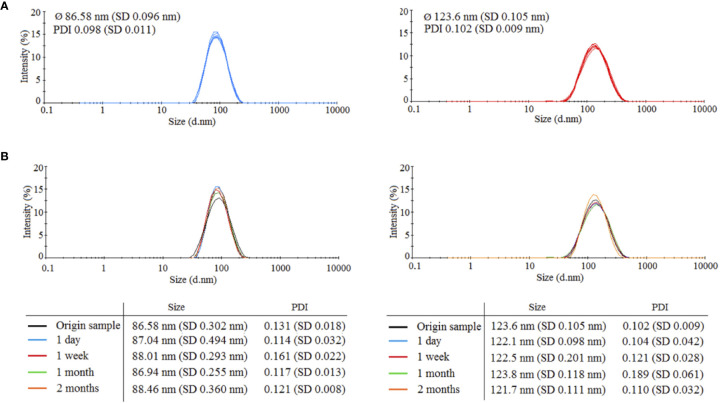
Characteristics of prepared 86.58 and 123.6 nm HANPs. **(A)** Size distribution of synthetized HANPs in 0.9% saline solution as determined by dynamic light scattering (DLS). **(B)** Stability of hyaluronic acid nanoparticles against irradiation by dose 17 Gy. Stability of HANPs was measured by DLS at times 1 day, 1 week, and 1 and 2 months after irradiation.

Two types of HANPs were prepared by covalent cross-linking of HA chains while using AAD as a cross-linking agent. HANPs (size 123.6 ± 0.105 nm) were first prepared by a simple cross-linking method. These HANPs were then used as an initial solution for double cross-linking procedure, the aim being to reduce their size (actual size was 86.58 ± 0.096 nm). Hydrodynamic diameters and polydispersity indices were monitored by DLS. Characteristics of both HANPs are shown **Figure 1A**. To verify the stability of HANPs against irradiation, the HANPs were exposed to IR (17 Gy). We observed no changes in hydrodynamic diameter of HANPs during 60 days after irradiation (**Figure 1B**).

**Figure 2:**
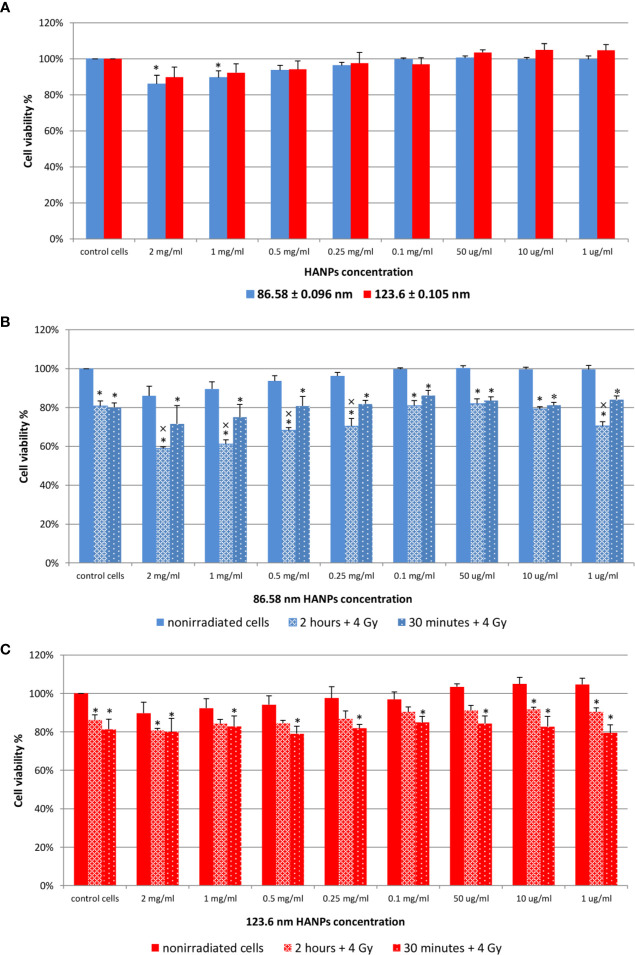
Effect of HANPs on cell viability. **(A)** Concentration-dependent effect on relative viability of J774.2 cell line after 24 h of incubation with 86.58 nm and 123.6 nm HANPs. **(B)** Viability of J774.2 after 2 h or 30 min preincubation with 86.58 nm and **(C)** 123.6 nm HANPs followed by 4 Gy irradiation. Each bar represents the mean ± 2× SEM. Asterisks (*) indicate significance differences at *p* ≤ 0.05 in comparison with untreated control and multiplication signs (×) indicate significance differences at *p* ≤ 0.05 in comparison with irradiated cells.

Next, relative viability of the J774.2 cell line after incubation with 86.58 or 123.6 nm HANPs was evaluated using WST-1 assay. **Figure 2A** shows the cell viability of 86.58 nm and 123.6 nm HANPs. The results indicated no significant cytotoxicity of 123.6 nm HANPs (confirmed by all statistical tests). Even at the highest concentration of 2 mg/ml, viability decreased only to 90% and nonsignificant increase in viability of cells compared to the untreated control was found from the 1–50 μg/ml concentrations. On the other hand, one-way ANOVA revealed significant differences between the control and 86.58 nm HANPs-treated groups. In the post-hoc analysis, only Fisher’s LSD test showed significantly reduced cell viability at higher concentrations ranging from 1 mg/ml to 2 mg/ml. Supported by these results, we can conclude that no observed cytotoxic effect was detected for 123.6 nm HANPs and cytotoxicity of 86.58 nm HANPs occurred only for concentration 0.5 mg/ml and higher.

The following experiment evaluated the effect of 2 h or 30 min preincubation with HANPs in combination with IR. The cells were exposed to a single dose of 4 Gy. Results are shown in **Figures 2B, C**. Neither concentration nor HANPs size showed a direct influence on radioprotective effect after preincubation. All irradiated cells had significantly decreased viability compared to the nonirradiated control, but differences were observed between groups depending on the size of HANPs and preincubation period used. A response difference was observed for preincubation time intervals when cells were incubated with 86.58 nm nanoparticles (**Figure 2B**). Higher NPs concentration in combination with IR caused significant decrease in cell viability. For concentrations ranging from 2 mg/ml to 0.25 mg/ml and for 1 µg/ml, the differences during 2 h of preincubation were significant not only when compared with untreated controls but also in comparison with irradiated cells. The highest viability of irradiated cells was observed at concentration 0.1 mg/ml for both preincubation intervals. Although the greater cell viability was achieved by 30 min preincubation for 86.58 nm HANPs, it was during longer preincubation that the 123.6 nm HANPs treatment improved the viability of cells (**Figure 2C**). At none of those concentrations of 123.6 nm HANPs used did viability decline in comparison with cells only irradiated, as seen in the case of 86.58 nm HANPs. Also, the greatest viability of irradiated cells was seen at the 0.1 mg/ml concentration point with these NPs. Based on the *in vitro* viability results described here, we decided for *in vivo* experiments to administer the highest possible HANPs concentration not having cytotoxic effect (i.e., 0.5 mg/ml) in experimental mice models *via* intratracheal instillation 30 min before irradiation.

### TGF-β and Mediators of Fibrosis Signaling Pathway Are Downregulated by HANPs Treatment

Cytokines have been reported as key features in the pathogenesis of RIPI resulting initially after irradiation and then simultaneously persisting and resolving into tissue damage. To evaluate the effect of HANPs on the inflammation and fibrosis phases in lung tissue, total protein levels and multiple cytokines were measured (**Table 1**). Time-course analysis of total protein levels in lung tissue demonstrated significantly increased level in the partial thoracic irradiated group (17 Gy) during the pneumonitis phase. This finding is consistent with the characteristics of RP, because damage to the integrity of vascular permeability leads to increased protein concentration in alveoli. By contrast, lungs treated with HANPs exhibited decreased levels of total proteins compared with the control group. Only in the group treated with 86.58 nm nanoparticles was a significant degree (*p* ≤ 0.05) of decrease observed.

**Table 1:** HANPs treatment modulates the concentration of total protein and cytokines in lung tissue lysates.

		Total protein [μg / ml]	IL-1β [pg / ml]	IL-6 [pg / ml]	TGF-β **[pg / ml]**	MCP-1 [pg / ml]	MIP-1 [pg / ml]	KC [pg / ml]	MMP-2 [pg / ml]	proMMP-9 **[pg / ml]**
**113** **day**	Control	2.52 ± 0.37	147.01 ± 9.75	102.68 ± 7.27	485.23 ± 18.64	104.37 ± 21.01	55.10 ± 4.94	4.27 ± 0.31	723.86 ± 20.38	13 068.10 ± 440.74
	17 Gy	2.71 ± 0.17 *	111.69 ± 3.30 *	52.03 ± 3.77 *	599.62 ± 49.61*	60.34 ± 8.60 *	34.57 ± 1.32 *	3.56 ± 0.34	1156.67 ± 164.29 *	10 421.42 ± 392.14 *
	86.58 nm + 17 Gy	2.12 ± 0.39 *	98.96 ± 4.40 * ×	65.28 ± 1.54 * ×	633.90 ± 11.76*	63.40 ± 3.14 *	34.50 ± 5.61 *	3.38 ± 0.49	964.61 ± 11.60 *	12 701.03 ± 1604.10 *
	123.6 nm + 17 Gy	2.26 ± 0.28	113.62 ± 2.32 * ○	71.54 ± 5.89 *	599.80 ± 31.38*	65.39 ± 12.34 *	50.74 ± 4.32 × ○	4.56 ± 0.22 × ○	813.24 ± 10.83 * ×	17 438.51 ± 1318.09 * × ○
	Control	2.35 ± 0.49	122.58 ± 1.25	96.23 ± 8.63	500.45 ± 30.66	109.89 ± 10.19	51.76 ± 5.05	3.92 ± 0.30	633.28 ± 11.00	9656.51 ± 243.25
**155 day**	17 Gy	2.23 ± 0.30	111.25 ± 11.29	40.47 ± 3.17 *	602.74 ± 11.92 *	66.61 ± 2.94 *	27.48 ± 2.85 *	3.13 ± 0.13 *	1252.83 ± 3.08 *	8063.81 ± 770.92 *
	86.58 nm + 17 Gy	2.38 ± 0.15	98.06 + 1.00 *	37.54 ± 4.74 *	875.43 ± 5.41 * ×	66.12 ± 6.17 *	28.45 ± 1.66 *	3.13 ± 0.32 *	1285.48 ± 1.47 *×	11 961.83 ± 247.14 *×
	123.6 nm + 17 Gy	2.31 ± 0.33	113.61 ± 3.78 *	45.65 ± 0.94 *	902.99 ± 24.22 * ×	62.23 ± 1.48 *	33.75 ± 3.93 *	3.66 ± 0.24	1027.20 ± 0.93 * × ○	20 575.71 ± 1045.02 * × ○
	Control	2.46 ± 0.28	86.25 ± 0.93	66.84 ± 5.79	605.49 ± 33.19	110.44 ± 10.81	97.58 ± 6.97	6.26 ± 0.27	557.02 ± 1.92	10 858.00 ± 2044.59
**190 day**	17 Gy	2.39 ± 0.26	312.35 ± 2.36 *	21.51 ± 2.28 *	904.11 ± 23.49 *	67.88 ± 1.87 *	74.98 ± 2.66 *	5.64 ± 0.15 *	1023.54 ± 1.28 *	5560.84 ± 131.56 *
	86.58 nm + 17 Gy	2.41 ± 0.37	168.17 ± 2.76 *×	--	661.87 ± 38.95 * ×	69.98 ± 4.28 *	87.66 ± 3.18 ×	4.64 ± 0.54 * ×	750.54 ± 0.47 * ×	10 882.15 ± 1949.15 ×
	123.6 nm + 17 Gy	2.01 ± 0.22 *	108.46 ± 1.32 * × ○	--	655.50 ± 5.14 * ×	55.23 ± 2.02 *	85.84 ± 2.87 ×	2.91 ± 0.21 * × ○	443.57 ± 5.50 * × ○	13 804.11 ± 3676.32 ×

Total protein of each sample and cytokine profile of IL-1β, IL-6, TGF-β, MCP-1, MIP-1, KC, MMP-2, and proMMP-9 from mixed group samples were measured in lung tissue. The assays were performed at days 113, 155, and 190 following lung irradiation (17 Gy). Each concentration level is presented as mean ± SD. Asterisks (*) indicate significance differences at *p* ≤ 0.05 in comparison with untreated control, multiplication signs (×) in comparison with irradiated cells, and small circles (○) in comparison with 85.68 nm HANPs + 17 Gy.

Results from cytokine analysis of the mixed group samples represented the trends in the temporal relationship between groups. At the time point 113 days after irradiation, levels of MCP-1 cytokine were significantly decreased compared to controls and their levels were similar in the irradiated-only group and both groups treated with HANPs. Contrariwise, the cytokine TGF-β significantly increased compared to controls, similarly in the irradiated-only group and both groups treated with HANPs. Cytokines KC and MIP-1 did not change significantly between the 123.6 nm HANPs group and the control, but these cytokines did exhibit decline in comparison with the irradiated-only group. The group treated with 86.58 nm HANPs showed decline compared with the control (significant only in the case of MIP-1) and the 123.6 HANPs groups. Other cytokines during this phase exhibited differences not only compared with the control but also among groups. The level of IL-1β decreased in the irradiated group and in the group treated with 123.6 nm HANPs (by 0.76- and 0.77-fold, respectively) compared to the control. The most significant depression, to 0.67-fold of the control, was in the group treated with 86.58 nm HANPs. This group differed from all the others. The cytokine IL-6 decreased most in the irradiated-only group (0.51-fold). Decrease was observed in both HANPs groups (to 0.64 and 0.70) compared with the control. For the mixed sample of collagen degrading MMP-2 and proMMP-9, however, concentration varied significantly between samples. The proMMP-9 is a precursor of MMP-9, and after insult (including by IR) it is cleaved from the pro- form to the active enzyme form (Atkinson and Senior, 2003). MMP-2 in the irradiated group was increased 1.6-fold while both HANPs irradiated groups showed tendencies to increase only minimally compared to the nonirradiated control (1.33- and 1.12-fold, respectively). Although the concentration of proMMP-9 was decreased in the irradiated group (0.8-fold vs. control), this protein was markedly increased (1.33-fold) relative to the control in the 123.6 HANPs group. The concentration in this group varied significantly in comparison with all other groups at day 113.

At the time point (day 155) for assessing the transition phase between RP and RF, the total protein levels revealed no significant differences among groups. Different trends were observed for the three pro-fibrotic proteins TGF-β, MMP-2, and proMMP-9. Markedly elevated concentrations of the protein TGF-β were observed in both HANPs groups compared to the nonirradiated and also to the 17 Gy irradiated-only group. The MMP-2 concentration increased similarly in both HANPs groups (2.0- and 1.62-fold, respectively) compared to the control, but significant difference in levels of pro MMP-9 versus control was observed between the HANPs groups (1.24-fold in the 86.58 nm HANPs group, but 2.13-fold in the 123.6 nm HANPs group). The trend exhibited in the 123.6 nm HANPs group was higher levels of proMMP-9 and only slight increase in MMP-2. Contrariwise, the trend in 17 Gy-irradiated group and group treated with 86.58 nm HANPs was different, levels of MMP-2 significantly increased and concentration of proMMP-9 increase slightly, even decreased in group only 17 Gy-irradiated. All other cytokines remained similar among all three treatment groups at this time point, which means they were significantly decreased compared with the control.

The time point representing radiation fibrosis (day 190) revealed that total protein levels in the lung treated with 123.6 nm HANPs decreased significantly (*p* ≤ 0.05) compared to the control. Also, cytokine concentration trends changed in this phase. Changes were again observed in the pro-fibrotic proteins TGF-β, MMP-2, and proMMP-9, as well as in IL-1β. The levels of IL-6 in both HANPs groups were below the detection limit. The most notable difference was between the 17 Gy-irradiated group and all three other groups. The results showed obvious elevation vis-à-vis the control in TGF-β (1.49-fold), MMP-2 (1.84-fold), and IL-1β (3.62-fold). That was in contrast to the level of proMMP-9, which was decreased relative to the control (0.51-fold) at this time point. The findings therefore reflect that application of HANPs of both sizes prior to irradiation modified the lung environment in a manner that markedly affected pro-fibrotic signaling.

### HANPs Changed Leukocytes Proportions in Peripheral Blood

**Figure 3:**
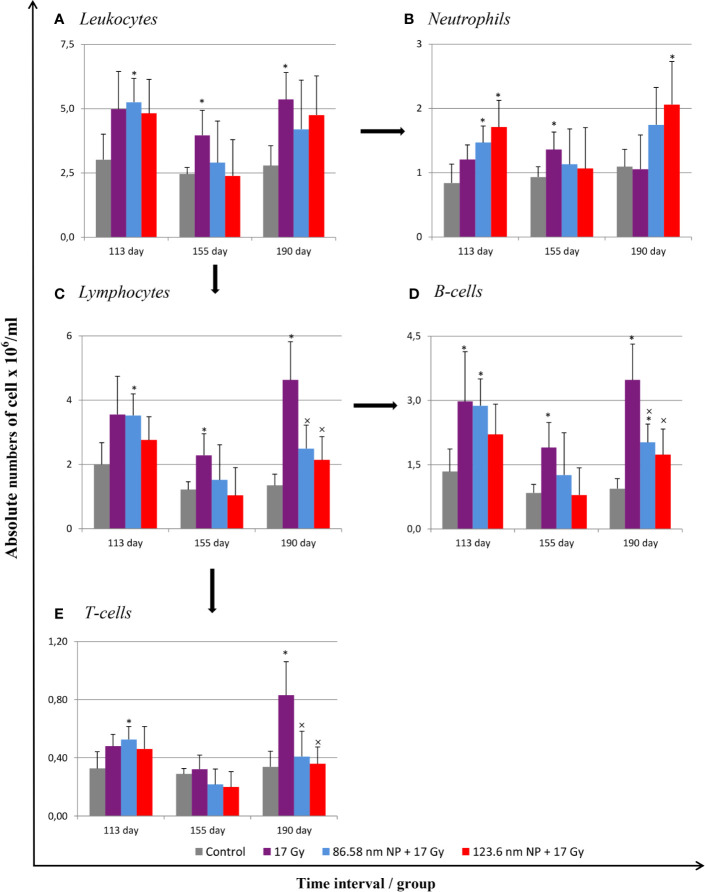
Changes in absolute counts of peripheral blood leukocytes after HANPs and IR treatments. **(A)** Analysis of peripheral blood leukocytes by hemoanalyzer. **(B)** Absolute counts of neutrophils and **(C)** lymphocytes in blood. Flow cytometry analysis of lymphocyte subpopulations of **(D)** B cells and **(E)** T cells. Analyses of peripheral blood were performed at days 113, 155, and 190 following lung irradiation (17 Gy). Each bar represents absolute number of cells presented as mean ± 2× SEM. Asterisks (*) indicate significance differences at *p ≤* 0.05 compared to the control group and multiplication signs (×) compared to the irradiated-only (17 Gy) group.

Radiation induces significant alterations in the absolute numbers of peripheral blood leukocytes. The analysis of cell populations in peripheral blood on the flow cytometer revealed changes in the representation of neutrophils as well as of both B and T lymphocytes. The results have shown that significant alternation in proportions of individual cell types depending on irradiation and HANPs treatment (**Figure 3**). At the time point corresponding with RP, day 113, only in the group treated with 86.58 nm HANPs was there a significant increase in total blood leukocyte counts, but the irradiated group and HANPs-treated groups varied significantly in the absolute numbers of their neutrophils and lymphocyte populations. In the 17 Gy partially irradiated group, the overall cell count response occurred prior to the lymphocytes response, mainly reflecting that the number of B lymphocytes had increased significantly compared with the control. By contrast, a significant increase in neutrophil count was identified in the 123.6 nm HANPs group. Significant changes were observed in the experimental group treated with 86.58 nm HANPs, as there was significant increase in all measured leukocyte populations and subpopulations with the exception of CD8^+^ cytotoxic T lymphocytes (data not shown). These trends were generally comparable with those seen also for the 123.6 nm HANPs group.

At day 155, representing the transition phase, there was—with one exception—decrease in total blood leukocyte counts in all populations and subpopulations compared with the pneumonic phase. The sole exception was significantly increased number of neutrophils in the 17 Gy-irradiated group. Moreover, this group exhibited significantly increased proportions of lymphocytes, and particularly numbers of B lymphocytes, relative to the control group. These values for groups of mice treated with HANPs of both size were similar to those of controls.

At the time point 190 days, however, there was a significant difference between the irradiated-only and HANPs-treated groups. During this fibrotic phase, the irradiated group displayed significant increase in lymphocyte number, represented by both B and T lymphocyte populations (cytotoxic CD8^+^, as well as helpers CD4^+^). Absolute numbers of lymphocytes in this group varied significantly not only in comparison to the control but also relative to those for HANPs-treated mice of both NP sizes. The effects observed in these groups were similar to those seen at the day 113 time point, when the cellular response had been more oriented to promoting increase in neutrophil count. Absolute numbers of neutrophils in the 17 Gy-irradiated group were therefore almost identical to those seen in the controls. These observations could suggest a role for HANPs in lymphocytes recruitment and promotion in the different stages of RIPI.

### Alterations in Lung Pathology and Leukocyte Infiltration in Lung Tissue From Mice Treated With HANPs

**Figure 4:**
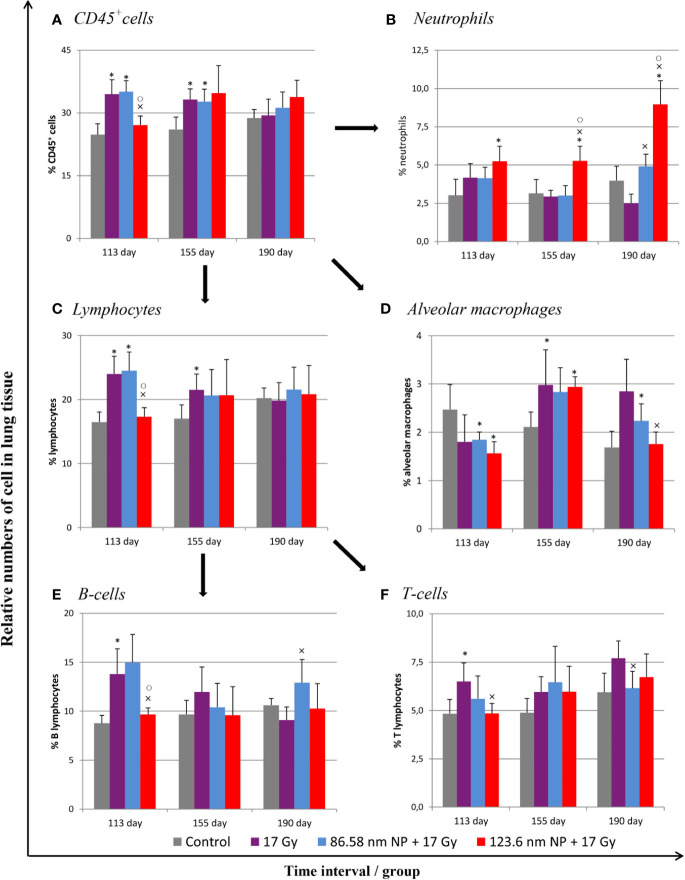
Flow cytometry analysis of lung tissue analysis after irradiation and HANPs application. **(A)** Immunophenotypization of leukocyte infiltration as measured by CD45^+^ cells, **(B)** neutrophils, **(C)** lymphocytes, and **(D)** alveolar macrophages. The lymphocytes population was subsequently analyzed for percentage representation of **(E)** B cells and **(F)** T cells subpopulations. HANPs were administered by intratracheal instillation prior to irradiation. Samples were prepared at days 113, 155, and 190 following lung irradiation (17 Gy). Each bar represents percentage of positive cells from total viable lung cells presented as mean ± 2× SEM. Asterisks (*) indicate significance differences at *p* ≤ 0.05 in comparison with control, multiplication signs (×) in comparison with the irradiated-only (17 Gy) group, and small circles (○) in comparison with the 85.68 nm HANPs + 17 Gy group.

**Figure 5:**
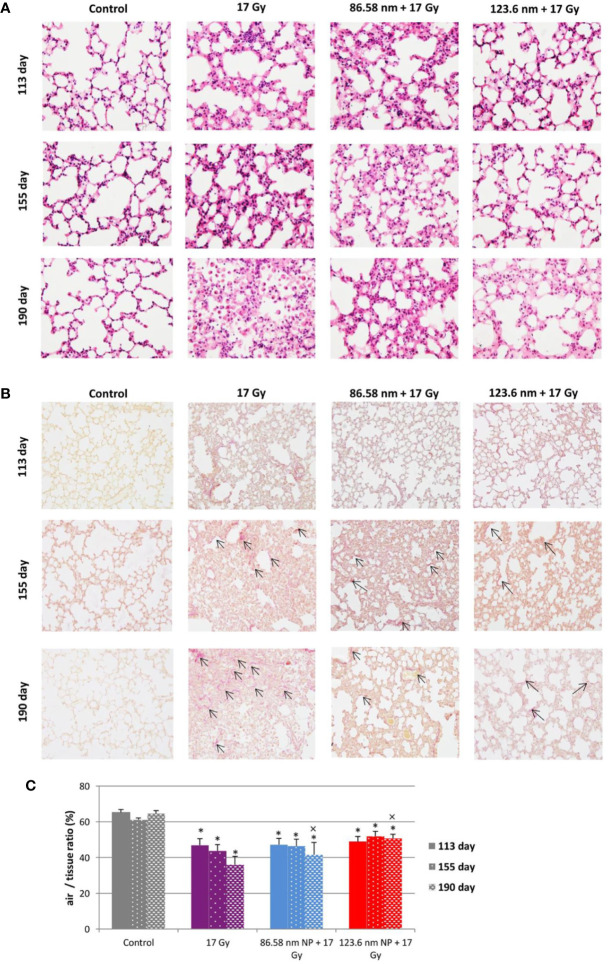
Lung tissue analysis. **(A)** Representative tissue sections of hematoxylin and eosin staining. **(B)** Representative tissue sections of picrofuchsin staining for collagen deposition in lung sections. **(C)** Quantitative analysis of airiness after radiation-induced damage to lung tissue and effect of HANPs treatment. Histology studies of lung tissue were performed at days 113, 155, and 190 following lung irradiation (17 Gy). Each bar represents absolute number of cells presented as mean ± 2× SEM. Original magnification 200×. Arrows indicate presence of collagen. Asterisks (*) indicate significance differences at *p ≤* 0.05 compared to the control group and multiplication signs (×) compared to the irradiated-only (17 Gy) group.

Because a major effect of radiation is injury to the alveolar epithelium and vascularity, the histopathological and cellular responses were analyzed. Histological staining of the lung tissue and analysis of CD45^+^ leukocyte infiltration was further quantified using flow cytometry of lung cell suspensions. At 113 days post-irradiation, significant decrease in tissue integrity was observed in all groups that had been irradiated. The air/tissue ratio was significantly decreased in all irradiated groups compared with the control (**Figure 5C**). Tissue sections showed markedly thickened alveolar walls, collapsed alveoli, and diffuse accumulation of inflammatory cells (**Figure 5A**). Applications of HANPs did not appear to have prevented damage to lung tissue. Although, deposition of collagen fibers were observed in the group only irradiated with 17 Gy, mainly around capillary vessels (**Figure 5B**). No difference in collagen deposition was seen between HANPs treated groups. Although inflammatory leukocytes infiltration (most especially by the lymphocytes population) was significantly dominant in the irradiated-only group and in the group where 86.58 nm HANPs and IR had been applied (**Figures 4A, C**), the percentage of leukocytes and lymphocytes in the group treated with 123.6 nm and IR, by contrast, did not vary significantly from the control. Moreover, analysis of this group revealed that the leukocytes population most significantly increased in the lung tissue after irradiation was that of neutrophils (**Figure 4B**). Lymphocytes and individual subpopulations play important roles in radiation-induced adverse effects within the lung. Significantly greater presence of B lymphocytes was observed in the irradiation-only group and the treatment using only the smaller-sized HANPs (**Figure 4E**). In the group with partial irradiation there also was recorded increased proportion of T lymphocytes in lung tissue (**Figure 4F**). The group treated with 123.6 nm HANPs and irradiated differed significantly from both other irradiated groups, but it had similar proportions of the various lymphocyte subpopulations as did the control. Another important innate immune cell population present in the alveoli is that of alveolar macrophages. The proportion of these cells decreased markedly after irradiation in the irradiated-only group and significantly in both groups treated with HANPs (**Figure 4D**). Complete cellular response in lung tissue was identical to cell response in peripheral blood during this phase. These data suggest a significant role of 123.6 nm HANPs in modulating lymphocytes response in lung tissue during the RP phase.

At the transition phase, represented by day 155, notable differences were observed between the 17 Gy irradiation and 86.58 nm HANPs treatment groups versus the 123.6 nm HANPs group. Similar to corresponding changes in peripheral blood, albeit with two exceptions, the percentage representations of all leukocyte populations and subpopulations decreased in comparison to the previous phase. The first exception was that the representation of alveolar macrophages in lung tissue was elevated during the transition phase. The second was that the lymphocyte proportion was slightly elevated due to an increasing of T cells in the 123.6 nm HANPs group. Also, this was the only group within which the numbers of neutrophils and B cells were the same as in the RP phase. On the other hand, the representation of B cells was shown to be most intensively decreased at day 155 (**Figure 4E**). Also, increased collagen deposition was observed in 86.58 nm HANPs treatment groups compared with the 123.6 nm HANPs group.

At the last time point, representing the fibrotic phase, the results manifested the most notable difference in cellular compartments of the irradiated lung between the irradiated-only group and groups treated with HANPs of both sizes. Not only was there observed in the lung tissue section significant improvement of tissue airiness and presence of fibrosis, due to collagen deposition, for the HANPs groups compared with the irradiated-only group (**Figure 5**), but also cellular responses between these groups clearly differed. Significant elevation in percentage numbers was observed in T lymphocyte and alveolar macrophage populations within the group treated with irradiation only. Compared with responses in peripheral blood, however, the representation of B cells was markedly reduced. Despite irradiation, and with neutrophils as the only exception (**Figure 4B**), the HANPs-treated groups showed no significant increase in cell representation during the fibrotic phase. In both HANPs groups the numbers of neutrophils increased significantly compared with the group only irradiated with 17 Gy. The 123.6 nm HANPs group even differed significantly in this population compared to all other groups. The results of our study point to an apparent effect of HANPs on the development of RIPI in both the RP and RF phases. The most evident modulation was observed at the time point representing the onset of RF in the lung tissue.

## Discussion

The hyaluronic acid molecule has been investigated intensively since its discovery (Meyer and Palmer, 1934) and to the present day. Due to its versatile properties in relation to its biocompatibility, biodegradability, non-toxicity, non-immunogenicity, and ubiquitous presence, as well as its characteristic metabolism pathways and signaling in organisms, this biopolymer has been used extensively, and especially in controlled-release and targeted drug delivery systems (Mattheolabakis et al., 2015; Huang and Huang, 2018). One of the most critical issues in radiobiology is search for suitable molecules, which can protect/modify response against destructive and damaging outcome of ionizing radiation exposure. Over the last few decades, many natural and synthetic compounds have been investigated for their potential as radioprotectors, mitigators, or therapeutics. In recent few decades, awareness in natural compounds as a potential source of radioprotectors has raised up, due to their ability to provide health benefits, less toxicity and common anti-inflammatory and antioxidant properties (Mun et al., 2018; Khodamoradi et al., 2020). Radioprotective effects to lung tissue have been demonstrated for numerous naturally occurring substances. Genistein, a soy isoflavonoids, provided mitigation of acute and late effects of lung tissue due to reduction of DNA damage in lung fibroblasts (Mahmood et al., 2011) or hesperidin, major flavonoid in lemons and sweet oranges, protected against oxidative stress damage caused by IR exposure by decreasing acute inflammatory pathways (Haddadi et al., 2017). Specifically, flaxseed demonstrated high protective effect against radiation fibrosis, inflammation and oxidative lung damage thought alternation in the TGF-β1 response (Lee et al., 2009). Also, alterations of radiation responses have been observed by endogenous occurring substances. Hormone melatonin significantly prevented against RIPI in the early pneumonic phase *via* a reduction in oxidative stress and the production of cytokines, such as TGF-β1 and TNF- α (Jang et al., 2015).

Although, possibilities for HA’s application in radiation biology have been limited since 1950, however, when it was proven that the HA molecule is radiosensitive, resolving in depolarization of primary chain and low fragment productions depending on absorbed dose of irradiation (Schoenberg et al., 1951). To the present day, only a few studies have addressed the potential for HA use in radiation biology. It has been confirmed that exogenous HA may serve as a radioprotective agent through TLR4 interaction in the intestine, that the HA molecule after IR exposure maintained antioxidant activity, that it significantly reduced radiation-induced inflammation and affected tissue hydration in patients with pelvic radiotherapy, and, in one study, that it improved radiation dermatitis (Liguori et al., 1997; Kim et al., 2008; Riehl et al., 2012; Cosentino and Piro, 2018). The present study is the first of its kind to confirm that intramolecularly cross-linked HA in nanoparticle form prevents ionizing radiation defragmentation. The study demonstrated long-term stability of synthetized HANPs after IR. In addition to stability against IR, the 86.58 nm and 123.6 nm HANPs exhibited great stability at various temperatures ranging from −80°C to 60°C, to enzymatic degradation, and to effects of model gastric juice (Kasparova et al., unpublished data). Results of the *in vitro* analysis confirmed that HANPs have no cytotoxic effect on the cell line. Choi et al. (2010) had demonstrated that the binding affinity of HANPs to the CD44 receptor is dependent on the NPs’ size. In the present study, we observed that the size of HANPs, but also the preincubation period and the IR insult, contributed to the final effect on cell viability (Choi et al., 2010). Consistent with our results, Gennari et al., (2019) confirmed a size-dependent effect of chitosan/HA nanoparticles on cell uptake and silencing efficiency in macrophages (Gennari et al., 2019). Recent studies of HANPs’ biodistribution and stability in organisms have demonstrated the most intensive tissues accumulation to be in liver, tumor, and lungs. Also, improved long-term stability and persistence of nanoparticles in the lung may be achieved by direct lung administration *via* intratracheal instillation (Choi et al., 2009; Kuehl et al., 2016).

Therefore, we decided to use these nanoparticles and investigate their effect on the course of the radiation-induced lung injuries that inevitably still accompany radiation therapy. The complexity of injury and contributions of various molecules and cell types in combination with direct radiation cytotoxicity pose complicated but appealing research challenges. Even though previous studies have not completely comprehended the full scope of the mechanisms involved, new treatment strategies have been employed. The most recent progress has been in nanomedicine. Radioprotective effects in preclinical models have been achieved through administering cerium oxide nanoparticles, manganese superoxide dismutase-plasmid/liposomes complexes, and nanoparticle formulations of Amifostine (Ethyol^®^) (Pamujula et al., 2005; Carpenter et al., 2005; Colon et al., 2009). Encouraged by these findings, we decided to test the effect of HANPs in an experimental model of RIPI.

The inflammatory phase of lung disease just after radiation is connected with the immediate response of hyaluronan and its metabolism during the initiation and progression (Li et al., 2000). Among all those molecules and cytokines investigated, TGF-β1 has been implicated as a key cytokine in the initiation, development, and persistence of both RP and RF (Finkelstein et al., 1994; Anscher et al., 1998; Rube et al., 2000). In the lung, TGF-β1 enhances the production of HA and gene expression for HAS2, HAS3, surface receptor CD44, and a receptor for HA-mediated motility (RHAMM) (Li et al., 2015; Ghatak et al., 2017). On the other hand, increased levels of HA in tissue have been shown to regulate response of the cells to TGF-β1 and HA/CD44/TGF-β1interaction is necessary for fibroblast proliferation (Webber et al., 2009; Meran et al., 2011). This reciprocal interaction between HA and TGF-β1 has been reported during tissue inflammation and fibrogenesis. These findings are consistent with our results, which demonstrated the crucial role of HANPs administration on the cytokines and their signaling pathways involved in RIPI. Importantly, TGF-β1 and irradiation are major stimuli that directly modulate the expression of MMP-2 and MMP-9, which are additional significant factors contributing to tissue fibrosis. MMPs are zinc enzymes responsible for the degradation of such ECM components as elastin, collagen, proteoglycans, laminin, and fibronectin during tissue remodeling processes. These processes are highly correlated with the response pattern in the early and late phase of injuries (Haiping et al., 2006). In our study, the increasing trend seen in the levels of these cytokines (TGF-β1, MMP-2, and pro-MMP-9) during courses of RIPI was confirmed only in the irradiated group). Nevertheless, the HANPs treatments significantly regulated the MMP-2 and MMP-9 balance in irradiated lung tissue. That, in turn, significantly affected maintaining the same structural integrity of pulmonary architecture. This was visible also in the histopathological section of lung tissue when compared with tissue from the irradiated group. That comparison was most striking on day 190.

Cytokine response in the tissue results from, but also is an effector of, the subsequent accumulation of immune cells and final tissue response. Our study revealed differential activation of leukocyte and macrophage cell subsets upon treatment. In the irradiated-only group, significant promotion of lymphocytic alveolitis development was apparent and depletion of resident alveolar macrophages was observed during the RP phase. With regard to fibrosis, this group was characterized most prominently by recruitment of macrophages and CD4^+^ T lymphocytes. These findings, as well as the cytokine profile findings are consistent with those from many other studies (Johnston et al., 2004; Chiang et al., 2005; Park et al., 2017). Lymphocytes have been reported as constituting a prominent feature of post-irradiation lung injury. The role of T lymphocytes has been discussed intensively, especially with regard to the plausible role of regulatory CD25^+^ lymphocytes (Wirsdörfer and Jendrossek, 2016). Therefore, the impact of B cells on the outcome of RIPI has not remained completely unrevealed. A study by Paun et al. (Paun et al., 2010) identified increased B cell gene expression after irradiation during intervals of RF, thus suggesting a possible new role of B cells in RIPI development. In the lung, B lymphocytes served in antigen presentation and as antibody-secreting plasma cells, but also in producing fibrogenic cytokines. Enhanced HA production has been noted to activate B cells producing these cytokines (Yoshizaki et al., 2008). Increased numbers of B cells were observed in peripheral blood during the present experiment, but only in the irradiated group.

It was predominantly 123.6 HANPs administration that seem to contribute most markedly to neutrophilic response. The possible role of neutrophils in RIPI is controversial. In their study, Johnston et al., (2004) suggested that neutrophils do not participate in RIPI devolvement within the mouse model. Other reports, however, have confirmed the infiltration of neutrophils during early time intervals after irradiation (Österreicher et al., 2004; Abernathy et al., 2015; Farhood et al., 2019). Neutrophils are the innate immune system’s effector cells recruited earliest to the site of pathology. Moreover, these polymorphonuclear cells have been shown to store MMP-2 and MMP-9 (pro-enzyme forms) in tertiary granules (Grommes and Soehnlein, 2011). A similar result of neutrophil activation due to induced nanotoxicity to lung tissue was reported by Sydlik et al., (2009) in their study of intratracheally instilled application of carbon nanoparticles. In our study, the increase in neutrophil counts was observed mainly in the group treated with 123.6 nm HANPs during RP and RF phases. Because no study to date has mentioned that application of HA molecule or HANPs induced lung toxicity, we expect that neutrophil recruitment was achieved due to exogenous presence of HANPs in tissue. This property of HA in relation to neutrophils already has been reported (Håkansson and Venge, 1985; McDonald et al., 2008). Furthermore, the crucial determinant of an induced biological effect is nanoparticle size. The results of our study imply moderate variances between groups treated with 86.58 nm HANPs versus 123.6 nm HANPs. Our results show that after irradiation the size of HANPs affected mainly neutrophils and B cells. On the other hand, cytokine levels, as well as the response of alveolar macrophages and T lymphocytes were comparable between the two HANPs groups but differed significantly from that of the irradiated-only group. Thus, understanding the interactions between nanomaterials and immune cells and their tissue distribution and mechanisms is important for the development of safe and effective nanomaterials for biomedical applications.

Overall, our study demonstrated that application of HANPs before irradiation provides substantial attenuation against RIPI. This was particularly significant during signal transduction processes that are relevant for the fibrotic phase. The complex of processes determining how HANPs impact the onset of radiation-induced cellular and molecular signaling patterns needs to be elucidated, and its confirmation remains an aim of our future work. In the end, we anticipate that this study can contribute to constructing a novel and useful vision for the field of radiation oncology that is based on nanomedicine modulation of lung toxicity and strives to develop efficient treatment strategies for patients.

## Data Availability Statement

The datasets generated for this study are available on request to the corresponding author.

## Ethics Statement

The animal study was reviewed and approved by Faculty of Military Health Sciences, Hradec Kralove, Czechia.

## Author Contributions

AL designed the experiments, participated in all the experiments, analyzed the data, wrote the manuscript and produced figures. ZS contributed to the conception and overall design of the study. JaP, KK, and MJ helped with performing of individual *in vivo* experiments and data analysis. JK, JiP, LK, and ZB were responsible for nanoparticle synthesis and characterizations. All authors contributed to the article and approved the submitted version.

## Funding

These studies were supported by the Ministry of Defence of the Czechia (long-term organization development plan Medical Aspects of Weapons of Mass Destruction of the Faculty of Military Health Sciences, University of Defence) and by the Ministry of Education, Youth and Sport, Czechia (Specific Research Project No: SV/FVZ201805) and by the OP RDE project, Strengthening interdisciplinary cooperation in research of nanomaterials and their effects on living organisms’ project no. CZ.02.1.01/0.0/17 048/0007421.

## Conflict of Interest

The authors declare that the research was conducted in the absence of any commercial or financial relationships that could be construed as a potential conflict of interest.
